# The well-being and treatment satisfaction of diabetic patients in primary care

**DOI:** 10.1186/1477-7525-8-67

**Published:** 2010-07-13

**Authors:** Esra Saatci, Gulruh Tahmiscioglu, Nafiz Bozdemir, Ersin Akpinar, Sevgi Ozcan, Hatice Kurdak

**Affiliations:** 1 Cukurova University Faculty of Medicine Department of Family Medicine, Adana, Turkey

## Abstract

**Background:**

The quality of life in patients with diabetes is reduced and emotional coping with the disease has great impact on patient well-being.

**Objectives:**

The aim of this study was to assess the psychological well-being and treatment satisfaction in patients with type 2 diabetes mellitus in primary care.

**Study Design and Setting:**

Patients (n = 112) with type 2 diabetes mellitus diagnosis for at least six months were enrolled. The Well-Being Questionnaire-22 and the Diabetes Treatment Satisfaction Questionnaire were used. Physical examination and laboratory investigations were performed.

**Results:**

The rates of the achieved targets were 32.1% for hemoglobin A_1c, _62.5% for cholesterol and 20.5% for blood pressure. The mean scores for the general well-being, depression, anxiety, positive well-being and energy were 44.40 ± 13.23 (range = 16-62), 12.65 ± 3.80 (range = 5-18), 10.57 ± 4.47 (range = 1-18), 12.00 ± 4.01 (range = 2-18), and 9.16 ± 2.47 (range = 2-12), respectively. The mean scores for the treatment satisfaction, perception for hyperglycemia and perception for hypoglycemia were 22.37 ± 9.53 (range = 0.00-36.00), 1.71 ± 1.59 (range = 0-6), and 0.51 ± 0.98 (range = 0-6), respectively. There were significant associations between the depression score and the educational status, compliance to diet and physical exercise, and diabetic complications; between the anxiety score and the educational status, glycemic control, compliance to diet and physical exercise; between the energy score and the educational status, compliance to physical exercise, and diabetic complications; between the positive well-being score and the educational status, compliance to diet and physical exercise, complications and type of treatment; between the general well-being score and the educational status, compliance for diet and physical exercise, and complications. Treatment satisfaction was significantly associated to the educational status, glycemic control and compliance to diet and physical exercise. A significant correlation was found between the treatment satisfaction and the well-being.

**Conclusions:**

Individualized care of patients with diabetes should consider improving the quality of life. Psychosocial support should be provided to the patients with type 2 diabetes and the negative effects of psychopathological conditions on the metabolic control should be lessened.

## Background

Diabetes is a chronic illness that requires continuing medical care and patient self-management education to prevent acute complications and to reduce the risk of long-term complications. Diabetes currently affects 246 million people worldwide and is expected to affect 380 million by 2025. The estimated prevalence of diabetes in the United States is 7.8% overall and 10.7% in the population over age 20 [1]. In 2002, visits to primary care physicians accounted for 62.7% of all office visits in the United States, and diabetes mellitus ranked third, accounting for 3.1% of illness-related diagnoses [2]. Diabetes is a frequent disease also in Turkey with a prevalence of 7.2% [3]. The quality of life (QoL) in patients with diabetes is reduced and it was well-shown that emotional coping with the diagnosis, daily treatment need and acute and chronic complications had great impact of the physical, psychological and social well-being of the patient with diabetes.

The aim of this study was to assess the level of psychological well-being and treatment satisfaction in patients with type 2 diabetes mellitus followed in primary care.

## Methods

### Sample

All patients (n = 112) with type 2 diabetes mellitus who admitted to Family Medicine Outpatient Clinic of Cukurova University Faculty of Medicine Department of Family Medicine from March 2007 to September 2007 were enrolled in this study. Inclusion criterion was to have diagnosis of type 2 diabetes mellitus for at least six months.

### Procedure and Data Collection

Three questionnaires were self-completed by the patients: sociodemographic (including questions on age, gender, educational status, body weight, height, duration of diabetes, acute and chronic complications, medications used, patient education on diabetes, other chronic conditions such as hypertension, hyperlipidemia, atherosclerotic heart disease, obesity etc, medications for the chronic conditions other than diabetes mellitus, compliance to diet and physical exercise, findings of the physical examination and laboratory investigations), Well-Being Questionnaire-22 (WBQ-22) and Diabetes Treatment Satisfaction Questionnaire (DTSQ). After physical examination including blood pressure measurement; ECG, blood laboratory tests (fasting blood glucose, postprandial blood glucose, glycosylated hemoglobin (HbA_1C_), total cholesterol, high density lipoprotein-cholesterol (HDL-C), low density lipoprotein-cholesterol (LDL-C), triglycerid, urea, and creatinine) and microalbuminuria in the spot urine were performed for all patients in the study.

### Questionnaires

#### WHO-Well-Being Questionnaire (WBQ-22)

The 22-item Well-Being Questionnaire (WBQ-22) [4-6] was originally designed in 1982 [7]. It was developed to provide a measure of mood, anxiety, and aspects of positive well-being for use in a WHO study evaluating new treatments for diabetes [8]. The instrument was initially developed with type 1 diabetes mellitus patients but has also been developed with type 2 diabetes mellitus patients. The items were derived from the psychological general well-being scale. It was designed to assess the patient's perception of general well-being and measures a component of quality of life known to be particularly relevant to patients with diabetes. The instrument was designed to assess the efficacy of new measures. The inclusion of a positive well-being dimension was designed to increase the sensitivity of the instrument. The positive well-being dimension was designed to assess psychological aspects of well-being, both negative and positive. Various versions of the questionnaire are already used in several diabetes programs across the world. The original instrument consisted of 28 items. The version used in this study, the WHO-WBQ [9], consists of 22 items with a Cronbach alpha value of 0.87 [10]. The WHO-WBQ-(22 item) is also used to construct a profile consisting of four subscales of depression, anxiety, energy, and positive well-being. Each item is scored on a 0 to 3 Likert-type scale, where "0" represents *not at all *and "3" represents *all the time*. The theoretical combined score range, therefore, extends from 0 (worst possible) to 66 (best possible). Ratings for the items are summed, after reversal where necessary. A higher score indicates more of the specific mood state. Savli and Sevinc [11] carried out the validity and reliability assessments of the scale in Turkey [12].

#### The Diabetes Treatment Satisfaction Questionnaire (DTSQ)

The Diabetes Treatment Satisfaction Questionnaire (DTSQ) was developed to assess the total diabetes treatment satisfaction and can be used in patients with type 1 or type 2 diabetes mellitus. It consists of eight items each rated on a seven-point Likert Scale. Six (items 1, 4-8) of them (item 1: satisfaction with current treatment; item 4: treatment convenience; item 5: flexibility of treatment; item 6: understanding of diabetes; item 7: continuity of treatment, and item 8: recommending treatment to others with diabetes), are summed to get treatment satisfaction score with a possible range of 0 (very dissatisfied) to 36 (very satisfied). Item 2 evaluates perceived frequency of hyperglycemia and item 3 perceived frequency of hypoglycemia and they are also rated on a seven point scale (0-6) same as other items but for these two items a score of zero indicates a lack of hyperglycemia or hypoglycemia while a higher score indicates a higher frequency. These two benchmark items were used for criterion validity testing [13].

### Ethics and Informed Consent

The study was approved by the Ethics Committee of Cukurova University Faculty of Medicine. Written informed consent was obtained from each patient in the study.

### Data Analysis

Data was installed and analyzed using SPSS for Windows 15.0 statistical pocket program. Mann-Whitney U, Kruskal Wallis, Pearson Chi-Square and Spearman's non-parametric correlation tests were used. We used Bonferroni test for multivariable comparisons. The level of significance was set as p < 0.05.

## Results

### Patient Demographics

Of patients 37 (33%) were male and 75 (67%) were female (Table 1). The mean age for males was 59.89 ± 10.40 years and the mean age for females was 55.60 ± 9.17 years (range = 35-85 years). Almost half of the patients were primary school graduates.

**Table 1:** Description of patient population (n = 112)

		Male n (%)	Female n (%)	Total n (%)
Age groups (years)	≤49	6 (16.2)	18 (24.0)	24 (21.4)
	
	50-54	5 (13.5)	20 (26.7)	25 (22.3)
	
	55-59	6 (16.2)	13 (17.3)	19 (17.0)
	
	60-64	6 (16.2)	13 (17.3)	19 (17.0)
	
	≥65	14 (37.8)	11 (14.7)	25 (22.3)
	
	Total	37 (33.0)	75 (67.0)	112 (100)

Education status	Illiterate	1 (2.8)	20 (27.4)	21 (19.3)
	
	Basic skills for reading-writing	5 (13.9)	13 (17.8)	18 (16.5)
	
	Primary school graduate	16 (44.4)	32 (43.8)	48 (44.0)
	
	High school	14 (38.9)	8 (11.0)	22 (20.2)
	
	Total	36 (33.0)	73 (67.0)	109 (100)

### Health indicators

The rates of achievement for the target levels of HbA_1c _(HbA_1c _< %7), cholesterol (<200 mg/dl), LDL-C (<100 mg/dl), triglyceride (<150 mg/dl), not having albuminuria, and blood pressure (<130/80 mmHg) were 32.1%, 62.5%, 32.1%, 33.0%, 83.3%, and 20.5%. There was no significant association between the blood test results (fasting and postprandial blood glucose, and lipid panel) and WBQ-22 scores of the patients (p > 0.05). Of our patients, 37.5% (n = 42) were obese and 38.4% (n = 43) were overweight. There was no significant relationship between patients' compliance to diet and physical exercise and body mass index (BMI) (p > 0.05 for both).

### WBQ-22 scale results

The mean scores for the general well-being, depression, anxiety, positive well-being and energy were 44.40 ± 13.23 (range = 16-62), 12.65 ± 3.80 (range = 5-18), 10.57 ± 4.47 (range = 1-18), 12.00 ± 4.01 (range = 2-18), and 9.16 ± 2.47 (range = 2-12), respectively. There was no significant association between gender and well-being scores (p > 0.05). Subscale scores of well-being showed improvement as the educational status of patient improved (Table 2).

**Table 2:** The Subscale Scores of WBQ-22 and Educational Status of Patients in the Study (n = 109)

Mean ± SD	Educational status					
	**Illiterate n = 21**	**Basic reading-writing skills n = 18**	**Primary school n = 48**	**High school n = 22**	**Total n = 109**	**p**

Depression	10.14 ± 3.1	10.11 ± 3.5	13.62 ± 3.2	15.63 ± 2.1	12.77 ± 3.7	0.0001

Anxiety	7.90 ± 4.5	9.55 ± 4.5	11.43 ± 4.5	12.45 ± 2.5	10.65 ± 4.4	0.004

Positive well-being	9.33 ± 3.4	10.00 ± 3.2	12.85 ± 3.9	14.90 ± 2.3	12.11 ± 3.9	0.0001

Energy	7.90 ± 2.8	7.94 ± 1.7	9.68 ± 2.3	10.45 ± 1.6	9.21 ± 2.4	0.0001

General well-being	35.28 ± 12.3	37.61 ± 11.9	47.60 ± 12.3	53.45 ± 7.0	44.76 ± 13.1	0.0001

There was a significant association between depression scores of patients who were illiterate and who were not (p = 0.0006) and between those who had basic skills of reading-writing and who were graduated from primary or high school (p = 0.0006). Anxiety scores of patients were also significantly different for these educational groups (p = 0.006 for illiterate patients and high school graduates; p = 0.036 for patients with basic skills of reading-writing and high school graduates). It was also true for the energy subscore (p = 0.018 for illiterate patients and high school graduates; p = 0.012 for patients with basic skills of reading-writing and primary school graduates; p = 0.0006 for patients with basic skills of reading-writing and high school graduates). It was similar for the positive well-being and general well-being subscores.

There was no significant association between the patients' body mass index and WBQ-22 subscores (p > 0.05). However, patients with better compliance for diet had better scores for depression, anxiety, positive well-being, and general well-being (p = 0.008, p = 0.014, p = 0.001, p = 0.002, respectively) and patients with better compliance for regular physical exercise had higher scores for all subscores (p = 0.0001 for each). Improvement in HbA_1c _levels were associated with less anxiety (p = 0.046). There was no significant association between the duration of diabetes and well-being scores (p > 0.05 for each). Patients' well-being was significantly associated with the presence or absence of diabetic complications (Table 3). Patients without any diabetic complications had better scores for depression, positive well-being, energy and general well-being (except for anxiety score) (p = 0.003, p = 0.003, p = 0.040, p = 0.018, respectively). The type of oral hypoglycemic agents (OHA) was significantly associated with positive well-being score (p = 0.024).

**Table 3:** The Subscale Scores of WBQ-22 and Diabetic Complications (n = 112)

Mean ± SD	Diabetic complications			
	**Yes (n = 30)**	**No (n = 82)**	**Total (n = 112)**	**p**

Depression	10.73 ± 4.0	13.35 ± 3.4	12.65 ± 3.8	0.003

Anxiety	9.66 ± 4.8	10.90 ± 4.3	10.57 ± 4.4	0.189

Positive well-being	10.03 ± 4.3	12.73 ± 3.6	12.00 ± 4.01	0.003

Energy	8.36 ± 2.6	9.46 ± 2.3	9.16 ± 2.4	0.040

General well-being	38.80 ± 14.7	46.45 ± 12.1	44.40 ± 13.2	0.018

### DTSQ scale results

Seven patients who had diabetes diagnosis for more than one year did not complete this scale as they were not using any medications due to various reasons. The mean scores for the treatment satisfaction, perception for hyperglycemia and perception for hypoglycemia were 22.37 ± 9.53 (range = 0.00-36.00), 1.71 ± 1.59 (range = 0-6), and 0.51 ± 0.98 (range = 0-6), respectively. There was no significant association between DTSQ mean total scores and patients' age, gender, BMI, the duration of diabetes, and the type of treatment (p > 0.05). DTSQ total scores showed a significant association with the patients' educational status (p = 0.0001) (Table 4). Patients with lower HbAıc levels had higher treatment satisfaction (p = 0.001) and patients with complications had lower treatment satisfaction (p = 0.0001).

**Table 4:** The total DTSQ Scores and Educational Status of Patients in the Study (n = 105)

Mean ± SD	Educational status					
	**Illiterate n = 21**	**Basic reading-writing skills n = 16**	**Primary school n = 46**	**High school n = 22**	**Total n = 105**	**p**

DTSQ total	18.09 ± 9.0	17.31 ± 8.8	24.65 ± 8.6	26.72 ± 8.9	22.65 ± 9.4	0.0001

There was no significant association between DTSQ total scores, DTSQ-2 or DTSQ-3 and compliance to diet and exercise (p > 0.05, for each). There was no significant association between DTSQ-2 and DTSQ-3 scores and the type of treatment (p > 0.05, for both).

### Comparing DTSQ and WBQ-22

There was a significant correlation between DTSQ total scores and WBQ-22 subscale scores (p = 0.0001 for each subscale score) (Table 5). There was significant correlation between DTSQ-2 and depression, anxiety, positive well-being, energy and general well-being scores (p = 0.002, p = 0.025, p = 0.005, p = 0.027, p = 0.004, respectively) (Table 6). There was significant correlation between DTSQ-3 and depression, energy and general well-being scores (p = 0.029, p = 0.036, p = 0.030, respectively) (Table 6).

**Table 5:** The Correlation between Subscale Scores of WBQ-22 and DTSQ Total Score (n = 105)

WBQ-22 subscales	DTSQ total score	
	**Correlation coefficient**	**p**

Depression	0.557	0.0001

Anxiety	0.464	0.0001

Positive well-being	0.503	0.0001

Energy	0.387	0.0001

General well-being	0.551	0.0001

**Table 6:** The Correlation Between Subscale Scores of WBQ-22 and DTSQ-2 and DTSQ-3 Scores (n = 105)

WBQ-22 subscales	DTSQ-2		DTSQ-3	
	**Correlation coefficient**	**p**	**Correlation coefficient**	**p**

Depression	-0.296	0.002	-0.210	0.029

Anxiety	-0.215	0.025	-0.145	0.134

Positive well-being	-0.270	0.005	-0.134	0.167

Energy	-0.213	0.027	-0.202	0.036

General well-being	-0.278	0.004	-0.209	0.030

## Discussion

Although it was reported that gender influenced well-being we could not find a significant association between gender and well-being [14-16]. This inconsistency may be due to the low number of patients in our study.

Although a number of authors [15,17] could not confirm the correlation between HbA1_c _and quality of life, a positive association has been reported in some cross-sectional surveys [18,19]. Savli et al found that there was a correlation between fasting and post-prandial blood glucose levels and anxiety score i.e. patients with diabetes worry with elevated blood glucose levels [11]. Van Tilburg et al revealed a significant positive relationship between depression and HbA1_c _in the type 1 group but not in the type 2 group [20]. On the contrary, Lustman et al showed in the meta-analytic review of the literature that depression was associated with hyperglycemia in patients with type 1 or type 2 diabetes [21]. We found a significant association only between HbA1_c _levels and anxiety scores. The poor association between metabolic control and well-being may be due to the fact that HbA1_c _represents the last four months where WBQ-22 the last 1-2 weeks.

In a study from primary care, mean HbA1_c _level was 7.6% and 40.5% of patients had values <7% [2]. Only 35.3% of patients were at or below target blood pressure (<130/85 mmHg) recommended by the American Diabetes Association (ADA) with only 74% below the Joint National Committee 7 (JNC 7) level for stage 1 hypertension (140/90 mmHg), only 43.7% had low-density lipoprotein cholesterol levels <100 mg/dL, and only 7.0% of patients met all three control targets [2]. These results are similar to those of NHANES 3 study (HbA_1c _level <7% in 42.3% of patients, mean value 7.8%) [22] whereas they are better than those reported in a recent retrospective study of general medicine and endocrinology clinics in academic medical centers from 2000-2002 (HbA_1c _<7% in 34% of patients, mean value 7.9%-8.1%) [23]. It was reported that only 37% of adult patients with diabetes achieved the goal of HbA1_c _level <7%, only 36% had blood pressure <130/80 mmHg, and only 48% had cholesterol level <200 mg/dl. Worse, only 7.3% of patients with diabetes could achieve these three targets [23]. In our study, 32.1% of patients achieved target levels of HbA_1c _(HbA_1c_<%7) and there was a significant association between HbA1_c _levels and anxiety score. The patients had less anxiety if their blood glucose control status was better. Our patients' cholesterol control was better than glycemic control with 62.5% of patients with total cholesterol level <200 mg/dl.

Among patients with diabetes, the benefits of regular physical activity have been well documented [24]. It was shown that restrictions on dietary freedom have a major negative impact on QoL [25]. We found a significant association between the compliance to diet and well-being subscale scores (except for the energy subscore). We also found significant association between regular physical exercise and all subscale scores of well-being. This can be related to better compliance for the disease or to being a good self-manager of this chronic condition. Patient's being able to achieve the recommended life style changes may contribute to the well-being of the diabetic patient.

Harris found that hypertension is an important risk factor for cardiovascular disease, nephropathy and retinopathy and clinical hypertension was present in 63% of patients [22]. Only 50.1% of patients with type 2 diabetes in NHANES III sample had blood pressures below 140/90 mmHg [22]. In our study, only 20.5% of our patients had adequate blood pressure control (<130/80 mmHg). This finding is consistent with the fact that despite significant Joint National Committee (JNC) efforts, a majority of patients are not reaching their blood pressure goals. A 2003 study conducted in eight managed care organizations in the United States concluded that less than 50% of plan members diagnosed with hypertension met their blood pressure goal (JNC 6). This conclusion held even after various educational and awareness campaigns were initiated [26]. In addition, data from the National Health and Nutrition Examination Survey (NHANES) revealed that only 36.8% of patients (including those undiagnosed) were at their target blood pressure [27].

The Third Report of the National Cholesterol Education Program (NCEP) Expert Panel on Detection, Evaluation and Treatment of High Blood Cholesterol in Adults (Adult Treatment Panel III) considers patients with diabetes to be at high risk of cardiovascular events and therefore recommends the same LDL-C goal as those for patients with established cardiovascular disease (CVD) (LDL-C < 100 mg/dl) [28]. In our study, 32.1% of patients achieved LDL-C target levels of 100 mg/dL compared with 15.4% of patients in the NHANES III study [22], 45% of patients in the Vermont Diabetes Information System Trial [29], and 43.7% of patients in the study by Spann et al [2].

Patients treated with insulin had higher depression and lower general well-being scores than patients treated with oral antidiabetic agents and diet [11]. We found a significant association between OHA and positive well-being. However, this finding should not be generalized as the number of patients administering insulin therapy was very few in our study.

Patients with a diabetes period of less than five years had lower depression scores compared to patients with a disease period of more than five years [11]. On the other hand, patients with a disease period of more than 20 years had lower energy and general well-being scores. Petterson et al found that patients with longer diabetes duration were generally more depressed and lacking in energy, positive well-being, and general well-being [15]. We could not find a significant association between the duration of diabetes and well-being scores probably due to low number of patients in our study.

Diabetic complications affect the quality of life in patients with diabetes leading to development of psychological disorders [11]. Akinci et al reported that patients with no complications reported significantly better overall health-related quality of life [30]. In a meta-analysis by De Groot, a significant association was found between depression and complications of diabetes [31]. Higher levels of depression were associated with increasing numbers of complications. Savli et al showed significant differences in well-being between patients with and without diabetic complications [11]. We found in our study that there was a significant association between well-being (except for anxiety) and diabetic complications. It was the expected situation that diabetic patients with complications had lower level for the well-being. The lack of significant association between diabetic complications and anxiety may be due to the unawareness or indifference of the diabetic patient.

### Treatment Satisfaction

In Diabetes and Territory Survey project, men were in general more satisfied than women about their treatment [32]. However, we could not find a significant association between gender and treatment satisfaction. We found a significant association between educational status and treatment satisfaction. Patients with better educational status had higher levels of treatment satisfaction.

Better results were achieved in patients treated with the oral hypoglycemic agents (OHA) mono-therapy if compared to those treated with insulin or combination therapy [32]. On the other hand, insulin was associated with greater improvements in treatment satisfaction [25]. We could not find a significant association between the type of treatment and treatment satisfaction probably due to the low number of patients using insulin. It was interesting that hypoglycemia and hyperglycemia perceptions were not significantly related to the type of treatment.

Finally, there was an inverse correlation between treatment satisfaction and HbA1_c _levels, indicating that the questionnaire could be informative to some extent regarding glyco-metabolic parameters [32]. We found a significant association between glycemic control and treatment satisfaction and between diabetic complications and treatment satisfaction.

Petterson et al found that the diabetes treatment satisfaction score correlated with general well-being [5,15]. The DTSQ treatment satisfaction score correlated most strongly with the general well-being score and least with the negative well-being subscale score [5]. We also found a significant correlation between treatment satisfaction and well-being in our patients.

Riazi et al reported that the item on the DTSQ measuring perceived frequency of hypoglycaemia correlated most strongly with negative well-being and least with positive well-being whereas the item measuring perceived frequency of hyperglycaemia correlated most with energy and least with positive well-being [5]. We found a significant correlation between DTSQ-3 score (hypoglycaemia) and WBQ-22 subscales of depression, energy and general well-being.

Diabetes is perceived to be significantly more difficult to manage than other common chronic conditions [33]. Inadequate patient skills, knowledge, and motivation about self-care, and physicians practice behaviors and settings are important determinants of adverse health outcomes [22]. Individualized care of patients with diabetes should consider both improving the quality of life and controlling risk for severe complications [34]. Further research will help us better understand the complex process-to-outcome relationships in diabetes care [2]. Psychosocial support should be provided to the patients with type 2 diabetes and psychopathological conditions including depression and anxiety should be treated and their negative effects on the metabolic control should be lessened [11]. Patients should be supported for being active participants in the management of their condition and balancing the biomedical and psychosocial outcomes [15].

### Limitations

Our study has some limitations. First of all the results were cross-sectional. We have low number of patients; particularly the ones under insulin therapy and all data were self-reported. Memory problems and misconceptions could not be excluded. We did not assess the relationship between income, sleeping, smoking, self-monitoring of blood glucose and well-being and treatment satisfaction. We could not compare the management of patients with diabetes in primary and tertiary level which was our aim in the beginning of the study planning. We could not assess the effect of diabetes patient education on well-being and treatment satisfaction as we did not have a formal patient education program and educated staff.

## Competing interests

The authors declare that they have no competing interests.

## Authors' contributions

ES contributed to the design, supervised data collection and analysis and wrote the manuscript. GT conducted data collection, entered data and contributed to the manuscript. NB contributed to the design, conducted statistical analysis and contributed to the manuscript. EA coordinated data collection and contributed to the manuscript. SO coordinated data collection and contributed to the manuscript. HK coordinated data collection and contributed to the manuscript. All authors read and approved the final manuscript.
